# ABC-Type Triblock Copolyacrylamides via Copper-Mediated Reversible Deactivation Radical Polymerization

**DOI:** 10.3390/polym14010116

**Published:** 2021-12-29

**Authors:** Fehaid M. Alsubaie, Othman Y. Alothman, Hassan Fouad, Abdel-Hamid I. Mourad

**Affiliations:** 1 National Center for Chemical Catalysis Technology, King Abdulaziz City for Science and Technology (KACST), P.O. Box 6086, Riyadh 11442, Saudi Arabia; 2 Department of Chemical Engineering, King Saud University, P.O. Box 800, Riyadh 11421, Saudi Arabia; 3 Applied Medical Science Department, Community College, King Saud University, P.O. Box 10219, Riyadh 11433, Saudi Arabia; menhfef@ksu.edu.sa; 4 Biomedical Engineering Department, Faculty of Engineering, Helwan University, Cairo 11792, Egypt; 5 Mechanical and Aerospace Engineering Department, College of Engineering, United Arab Emirate University, Al Ain P.O. Box 15551, United Arab Emirates; ahmourad@uaeu.ac.ae; 6 National Water and Energy Centre, United Arab Emirate University, Al Ain P.O. Box 15551, United Arab Emirates; 7 Mechanical Design Department, Faculty of Engineering, Helwan University, Cairo 11795, Egypt

**Keywords:** triblock copolyacrylamides, macroinitiator, controlled polymerization, radical polymerization, undesirable side reaction, dispersity, chain extensions

## Abstract

The aqueous Cu(0)-mediated reversible deactivation radical polymerization (RDRP) of triblock copolymers with two block sequences at 0.0 °C is reported herein. Well-defined triblock copolymers initiated from PHEAA or PDMA, containing (A) 2-hydroxyethyl acrylamide (HEAA), (B) N-isopropylacrylamide (NIPAM) and (C) N, N-dimethylacrylamide (DMA), were synthesized. The ultrafast one-pot synthesis of sequence-controlled triblock copolymers via iterative sequential monomer addition after full conversion, without any purification steps throughout the monomer additions, was performed. The narrow dispersities of the triblock copolymers proved the high degree of end-group fidelity of the starting macroinitiator and the absence of any significant undesirable side reactions. Controlled chain length and extremely narrow molecular weight distributions (dispersity ~1.10) were achieved, and quantitative conversion was attained in as little as 52 min. The full disproportionation of CuBr in the presence of Me_6_TREN in water prior to both monomer and initiator addition was crucially exploited to produce a well-defined ABC-type triblock copolymer. In addition, the undesirable side reaction that could influence the living nature of the system was investigated. The ability to incorporate several functional monomers without affecting the living nature of the polymerization proves the versatility of this approach.

## 1. Introduction

The homo-polymers and copolymers of acrylamide-based monomers have been employed in various applications [1,2,3,4,5]. Although the free-radical polymerization of acrylamide derivatives has been thoroughly investigated, developed controlled free-radical polymerizations have recently been utilized to produce new polymeric materials with unique properties. A few years ago, reversible deactivation radical polymerization (RDRP) started to attract great attention. The RDRP of acrylamide-based monomers has proven challenging when water is used as the solvent. Usually, in aqueous solutions, the rate of the RDRP process is rapid, resulting in termination events. This has been attributed to the lack of equilibrium between the dormant species and the active radical species. During the polymerization processes and the activation and deactivation steps, equilibrium is key to attaining the linear polymeric chains’ growth. The polymer research community has developed techniques to achieve fast equilibrium between dormant and active chain species, including nitroxide-mediated polymerization (NMP) [6,7], reversible addition–fragmentation chain transfer (RAFT) [8,9], atom transfer radical polymerization (ATRP) [10,11,12], in situ polymerization [13,14] and single-electron transfer living radical polymerization (SET-LRP) [15]. 

The mechanism of copper-mediated living radical polymerization (CM-LRP) proposes the equilibrium between propagating species and alkyl halides that can be mediated by Cu–ligand complexes [16,17,18,19,20]. To achieve better control of CM-LRP, a mixture of water and methanol media should be used [21]. It was reported that the polymerization of acrylamide-based monomers in water is rather difficult due to undesirable termination reactions that cause a loss of chain-end fidelity [21,22,23]. The aqueous Cu-mediated LRP of acrylamides has been shown to be problematic with regard to the control of the polymerization when water is employed as the only solvent at ambient temperature [24,25]. Furthermore, in the literature, few publications have reported controlled block copolymerization, and they are limited to just diblock. Brittain et al. [21] used different copper salts to support the aqueous ATRP of dimethylacrylamide. They concluded that the addition of the Cu salt complex to the amide group of the chain ends stabilized the radicals, resulting in an increase in the radical concentration and side reactions. 

SET-LRP is an RDRP process whereby polymers with high end-group functionality can be synthesized. In this process, the key step is the disproportionation of Cu(I)Br in the presence of nitrogen-containing ligands, as this produces both the activator Cu(0) and the deactivator Cu(ΙΙ)Br_2_ [15]. The equilibrium constant for the disproportionation depends mainly on the nature of the solvent and ligand. The equilibrium constant is noticeably higher in the presence of Me_6_TREN aqueous media than in organic solvents [26,27,28,29].

Whittaker et al. [29] applied Cu(0)-mediated polymerization to formulate high-order multiblock copolymers at atmospheric temperature in DMSO. The copolymers (consisting of four acrylates) were synthesized via sequential monomer addition to build multiblock copolymers model P(MA-*b*-MA) homo-polymer and P(MA-*b*-nBuA-*b*-EA-*b*-2EHA-*b*-EA-*b*-nBuA) to allow each block to have very discreet blocks (ideally two monomer units). Interestingly, this shows that full conversion of the monomer can be reached, which illustrates the effectiveness of this technique. The molecular weight distributions remained fairly narrow (Ð ~1.2) after monomer additions (24 h per block), proving that the polymerization is highly controlled without any purification steps. Later, the authors employed a similar approach to synthesize a decablock copolymer. However, they obtained molecular weight distributions (1.72) without quantitative conversion [30].

Later, the Whittaker group [31,32] utilized a similar technique to synthesize higher-molecular-weight block homo-polymers and copolymers of different acrylates (each block DPn ≈ 100). Narrow dispersities (<1.2) were achieved up to the sixth block, while the monomer conversion rate ranged from 92 to 100%. Moreover, multiblock glycopolymers (the degree of polymerization (DP) = 2 for each block, (mannose)_2_-(glucose)_2_-(mannose)_2_-(glucose)_2_-(mannose)_2_-(glucose)_2_) were prepared [33]. The copolymerization was performed in DMSO at room temperature, with a total reaction time of 46 h, resulting in good control over the molecular weight distributions.

Another approach to the synthesis of multiblock copolymers is to employ RAFT as a means of polymerization. The synthesis of an icosablock (20 blocks) has been achieved [34,35], with each block consisting of an average of three monomer units composed of three types of acrylamide monomer, namely, N, N-dimethylacrylamide (DMA); 4-acryloylmorpholine (NAM); and N, N-diethylacrylamide (DEA). Although this is a significant addition to the copolymerization field, its utility is offset by the high reaction temperature (70 °C). 

The advantage of the full disproportionation of Cu(I)Br/Me_6_TREN in water prior to monomer and initiator addition to synthesize both homo- and multi-block copolymers was previously exploited [36,37]. The most obvious advantage of these works is that the quantitative conversion was achieved in minutes, with final dispersity (Ð < 1.15). Interestingly, suppression of the hydrolysis reaction by employing lower reaction temperatures (i.e., ice/water bath) allowed for the copolymerization to proceed without appreciable loss of the polymerization control [37,38]. However, the synthesis of a multiblock copolymer (macromolecular alkyl bromide) was only chain extended from a NIPAM monomer, as this is the model monomer. In addition, the rate of ω-Br chain-end loss was pronounced in tertiary acrylamides relative to secondary acrylamides. Since N-hydroxyethyl acrylamide has been widely utilized in industrial and biological applications [39,40,41,42], the ability of poly N-hydroxyethyl acrylamide (PHEAA) as a macroinitiator to initiate other acrylamide monomers has recently attracted more attention. Narumi and his co-workers [43] employed the ATRP technique to produce block copolymers from PHEAA as a prepolymer. Although narrow MWDs were achieved, they were limited to just diblock. In addition, the controlled polymerizations were conducted in ethanol/water mixtures at ambient temperature, which could prevent their use in applications where pure water as the solvent is required. Furthermore, the integrity of the diblock compositions and one-pot procedure were not reported.

Herein, the one-pot polymerization procedure was exploited to synthesize a well-defined triblock copolymer from PHEAA as a macromolecular model ABC, containing (A) 2-hydroxyethyl acrylamide (HEAA); (B) *N*-isopropylacrylamide (NIPAM); and (C) *N*, *N*-dimethylacrylamide (DMA) segments, utilizing Cu(0)-mediated RDRP. PDMA was also employed as a macroinitiator to initiate another sequence of triblock copolymers (CBA). Pure water was utilized as the solvent to produce the different block sequences below ambient temperature. The copolymerizations proceeded in a controlled manner (Ð = 1.10), and full conversions were obtained within ~1 h, as evidenced by ^1^H NMR and SEC analyses. Likewise, prior disproportionation in situ was also exploited toward the ultrafast one-pot synthesis of sequence-controlled triblock (ABC model) copolymers of different acrylamides, allowing successive chain extensions and end-group fidelity. In addition, the undesirable side reaction was investigated.

## 2. Materials and Methods

### 2.1. Materials

HEAA (97%), NIPAM (97%) and DMA (99%) were obtained from Sigma-Aldrich (St. Louis, MO, USA). Prior to use, the monomers were filtered by injection over a column filled with alumina to eliminate inhibitors. HPLC-grade H_2_O (VWR international, LLC, Radnor, PA, USA) was used as the solvent for all reaction processes.

The water-soluble initiator (WSI), namely, dihydroxypropane-3-oxy-(2-bromo-2-methylpropionyl), and tris [2-(dimethylamino) ethyl]amine (Me_6_TREN) were synthesized according to previously reported procedures [34]. Copper bromide (98%, Sigma-Aldrich) was repeatedly purified with acetic acid and ethanol.

### 2.2. Instruments and Analysis

The proton nuclear magnetic resonance spectrum was recorded on a JEOL NMR spectrometer (600 MHz), and the NMR solvent was D_2_O as a locking agent. The monomer conversions for the reaction of HEAA, NIPAM and DMA polymerization were obtained by evaluating the integrals of the peaks corresponding to the vinyl protons, as well as those attributed to the N-methylene signal, the isopropyl methine proton and the methyl signal of DMA, respectively.

Size-exclusion chromatography (SEC) was utilized in the Agilent N29812 system (Agilent, Santa Clara, CA, USA), using dimethylformamide (DMF) containing 5 mM NH4BF4 as the mobile phase at 45 °C. The system was equipped with ultraviolet, refractive index and viscometer detectors, as well as an autosampler, a PLgel 5 μm MIXED-D column (300 × 7.5 mm^2^) and a PLgel 5 μm Guard column (50 × 7.5 mm^2^). In addition, an Agilent 1260 infinity isocratic pump with a maximum pressure of 600 bar was used.

Commercially available narrow linear polymers (PMMA) in the range of 200 to 1.0 × 10^6^ g mol^−1^ were used to calibrate the system of reactions. All samples were passed through a 0.45 μm polytetrafluoroethylene (PTFE) filter. All reactions were performed under a N_2_ blanket, and dissolved air in the solution was removed by bubbling for 15 min. Then, the removed air left the solution throughput point existing in the top of the glassware, and we employed the Schlenk technique.

### 2.3. Synthesis of ABC-Type Triblock Copolymer by Aqueous Cu(0)-Mediated Polymerization at 0.0 °C

The disproportionation reaction of CuBr in the presence of Me_6_TREN ligand was conducted as reported in the literature [30]. H_2_O (1 mL), dihydroxypropane-3-oxy-(2-bromo-2-methylpropionyl) (60 mg, 0.00025 mole) and acrylamide monomer (2.5 mmol) were added to a clean Schlenk tube, and the solution was deoxygenated by bubbling with nitrogen for 15 min. The deoxygenated initiator/monomer solution was then injected into the disproportionation reaction Schlenk tube, which had already been immersed in the ice/water bath. The polymerization Schlenk tube was then stirred at 0.0 °C for a certain period of time. A sample was taken for conversion analysis before the addition of deoxygenated aqueous solutions of the second and third monomers at full conversion of the first and second block. Samples of the reaction mixtures were removed by a degassed syringe for ^1^H NMR and SEC analysis.

## 3. Results and Discussion

Well-defined multiblock copolymers of different acrylamide derivatives were synthesized via SET-LRP in water. However, the multiblock copolymers were only initiated from PNIPAM, as this monomer is the model monomer [36,37]. To the best of our knowledge, only very few publications have reported the controlled diblock copolymerization of acrylamides initiated from PHEAA or PDMA via RDRP techniques. In addition, ABC-type triblock copolymers initiated from a PHEA-based macroinitiator have not yet been reported. Changing the sequence of blocks is particularly important due to the chemical structures and properties. Therefore, the ability of PHEAA and PDMA to initiate block copolymers was investigated.

### 3.1. Investigating the Potential for ABC-Type Triblock Copolymer Synthesis via Chain Extension of PHEAA or PDMA Macroinitiator

**Scheme 1:**
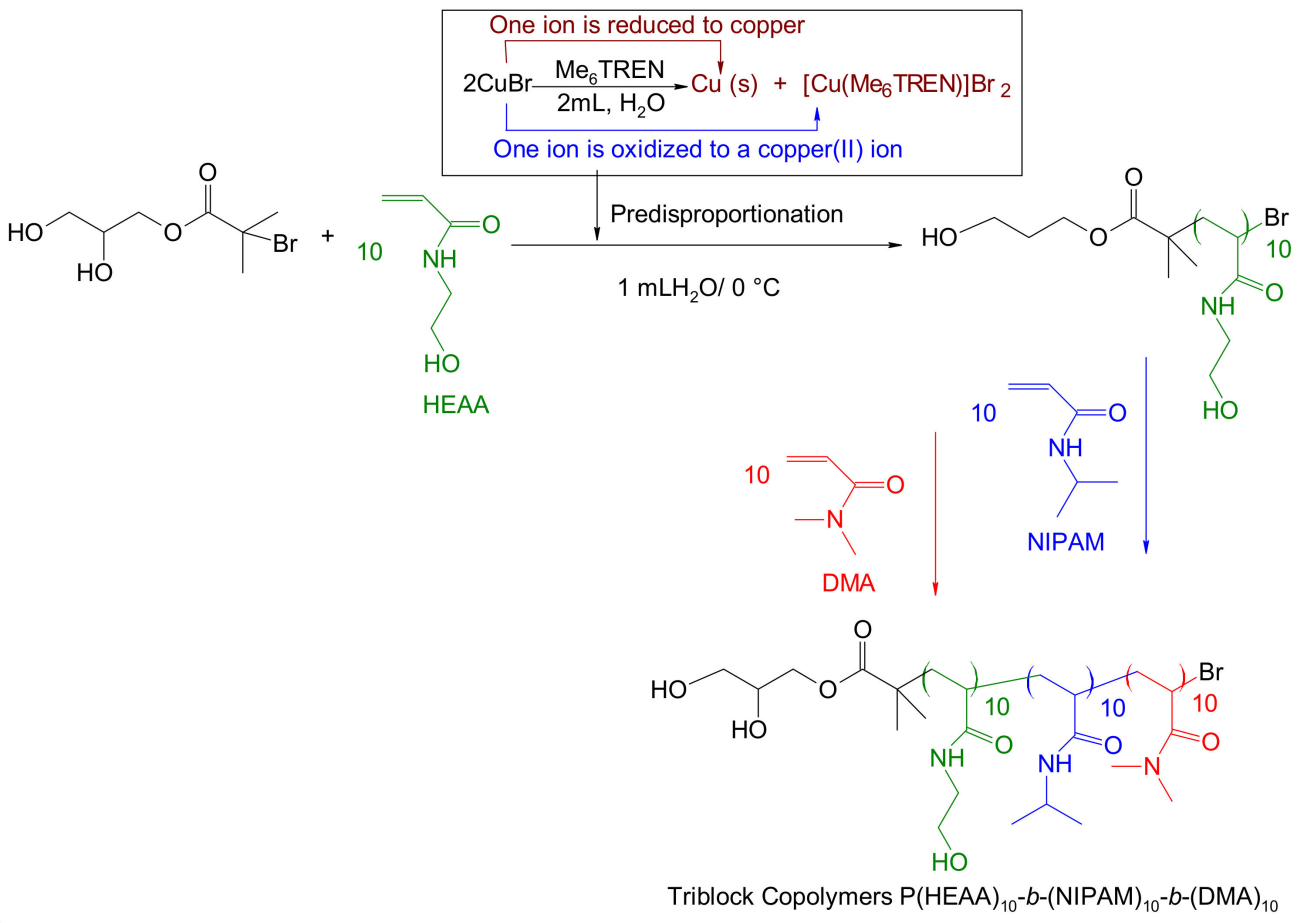
Synthesis of triblock copolymers composed of HEAA, NIPAM and DMA by iterative Cu(0)-mediated polymerization in H_2_O at 0.0 °C via prior disproportionation. Conditions: [WSI]:[monomer]:[CuBr]:[Me_6_TREN] = 1:10:0.4:0.4.

The synthesis of two different block sequences of model triblock copolymers P(HEAA)10-*b*-(NIPAM)10-*b*-(DMA)10 and P(DMA)10-*b*-(NIPAM)10-*b*-(HEAA)10, with each block comprising ten monomer units (DP 10), was targeted. The Cu(0)-mediated polymerization method was utilized in pure water at 0.0 °C, offering a new route to biological applications. The direct disproportionation of CuBr in the presence of Me_6_TREN was exploited to generate Cu(0) and CuBr_2_ prior to iterative Cu(0)-mediated polymerization processes, as presented in Scheme 1.

**Figure 1:**
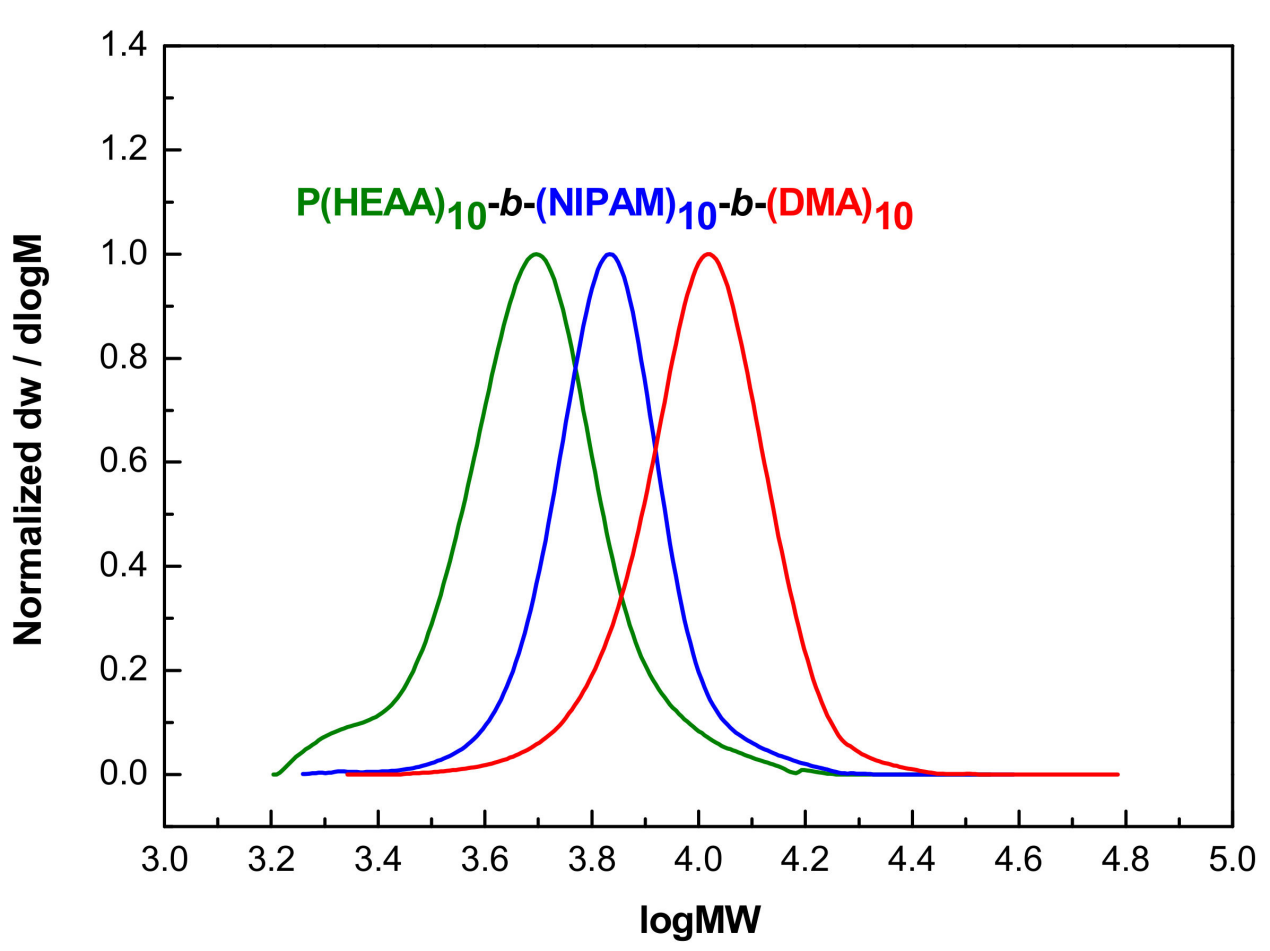
The evolution of molecular weights of successive block extensions of HEAA, NIPAM and DMA by iterative Cu(0)-mediated polymerization.

After full disproportionation of CuBr, the first aliquot of monomer was allowed to polymerize under nitrogen protection. Interestingly, the full monomer conversion of the first block was attained in 20 min according to the total disappearance of vinyl groups confirmed by ^1^H NMR analysis. The molecular weight distributions of the first block were extremely narrow, with dispersity (Ð = 1.09) (Table 1, Figure 1), and no issues with the purity of both the initiator and ligand to conduct the polymerization were found. 

In the direction of our target and in order to avoid any side reaction to the end chains of the first block during NMR and SEC measurements, we repeated the polymerization, and the second aliquot of the NIPAM monomer was directly added after 20 min. As hoped, ^1^H NMR analysis confirmed that the quantitative conversion of the second block was attained in 40 min, and SEC showed successful chain extension with dispersity (Ð = 1.08). The fast, controlled and quantitative conversion of the first and second block without the need for a purification step encouraged us to proceed with copolymerization. Importantly, prior to proceeding further, every chain extension cycle was precisely identified in terms of time and conversion. Consequently, the triblock copolymer was obtained via iterative chain extension in a very short reaction time (~1.5 h), negating the purification between monomer additions.

**Figure 2:**
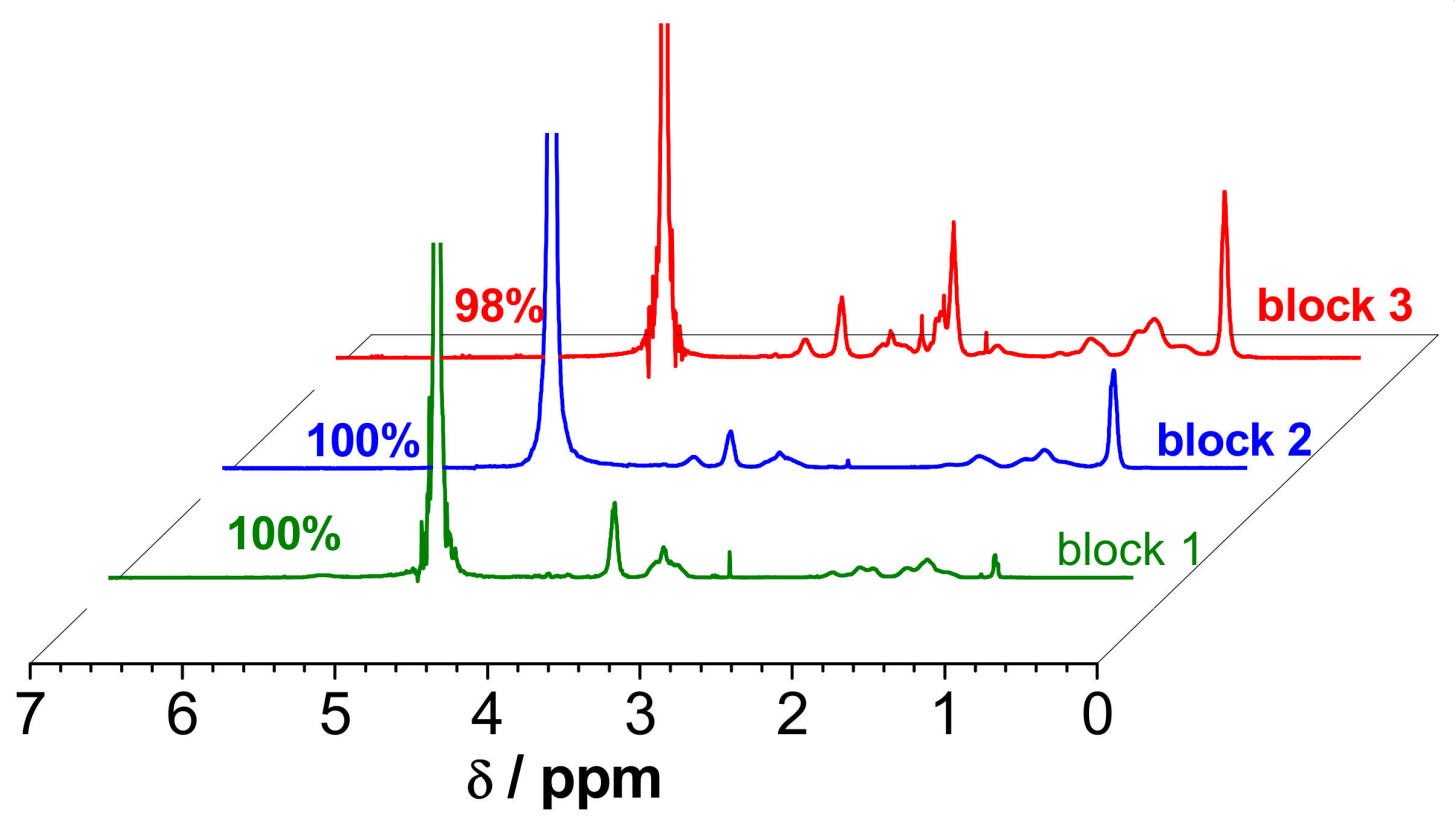
^1^H nuclear magnetic resonance (NMR) spectra (D_2_O) for triblock copolymers of HEAA, NIPAM and DMA and by iterative Cu(0)-mediated polymerization.

Quantitative conversions were confirmed by the ^1^H NMR analysis of each block according to the integration of the vinyl protons (~6.50–5.70 ppm) of the monomer with the N-methylene signal of HEAA (NH(–CH_2_–) (~3.3 ppm)), in the case of the second block, with the isopropyl protons of NIPAM (-CH (CH_3_)_2_) (~3.50–3.90 ppm) and with the methyl signal of DMA (N(CH_3_)_2_ (~3.0 ppm)) (Figure 2). Moreover, the molecular weight distributions of the triblock copolymers remained extremely narrow throughout the monomer additions, with a final dispersity of <1.09. These data demonstrate the level of sequence control obtained in one pot of such a new ABC-type triblock copolymer, reflecting the robustness and versatility of this method.

**Scheme 2:**
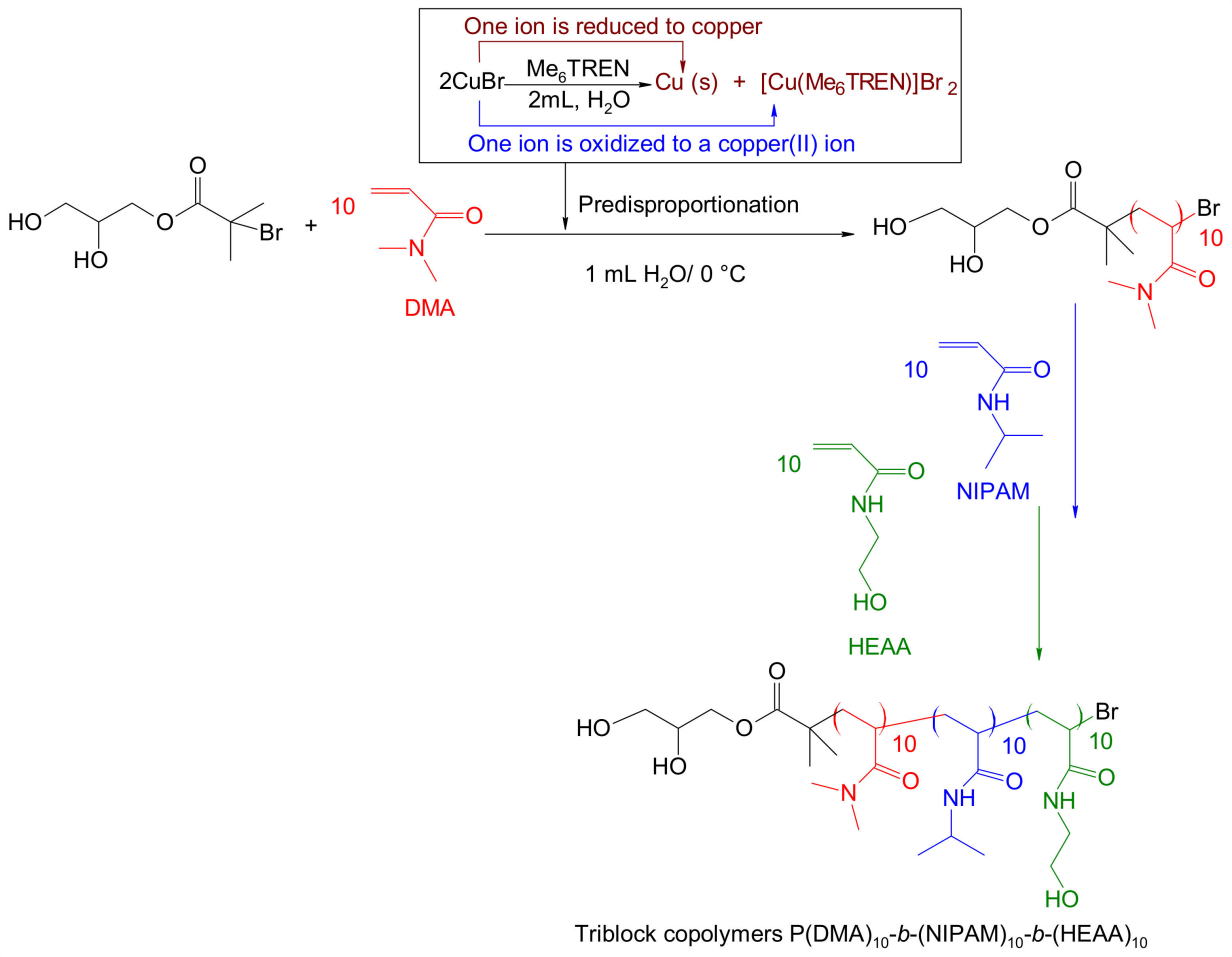
Synthesis of triblock copolymers composed of DMA, NIPAM and HEAA by iterative Cu(0)-mediated polymerization in H_2_O at 0.0 °C via prior disproportionation. Conditions: [WSI]:[monomer]:[CuBr]: [Me6TREN] = 1:10:0.4:0.4.

**Figure 3:**
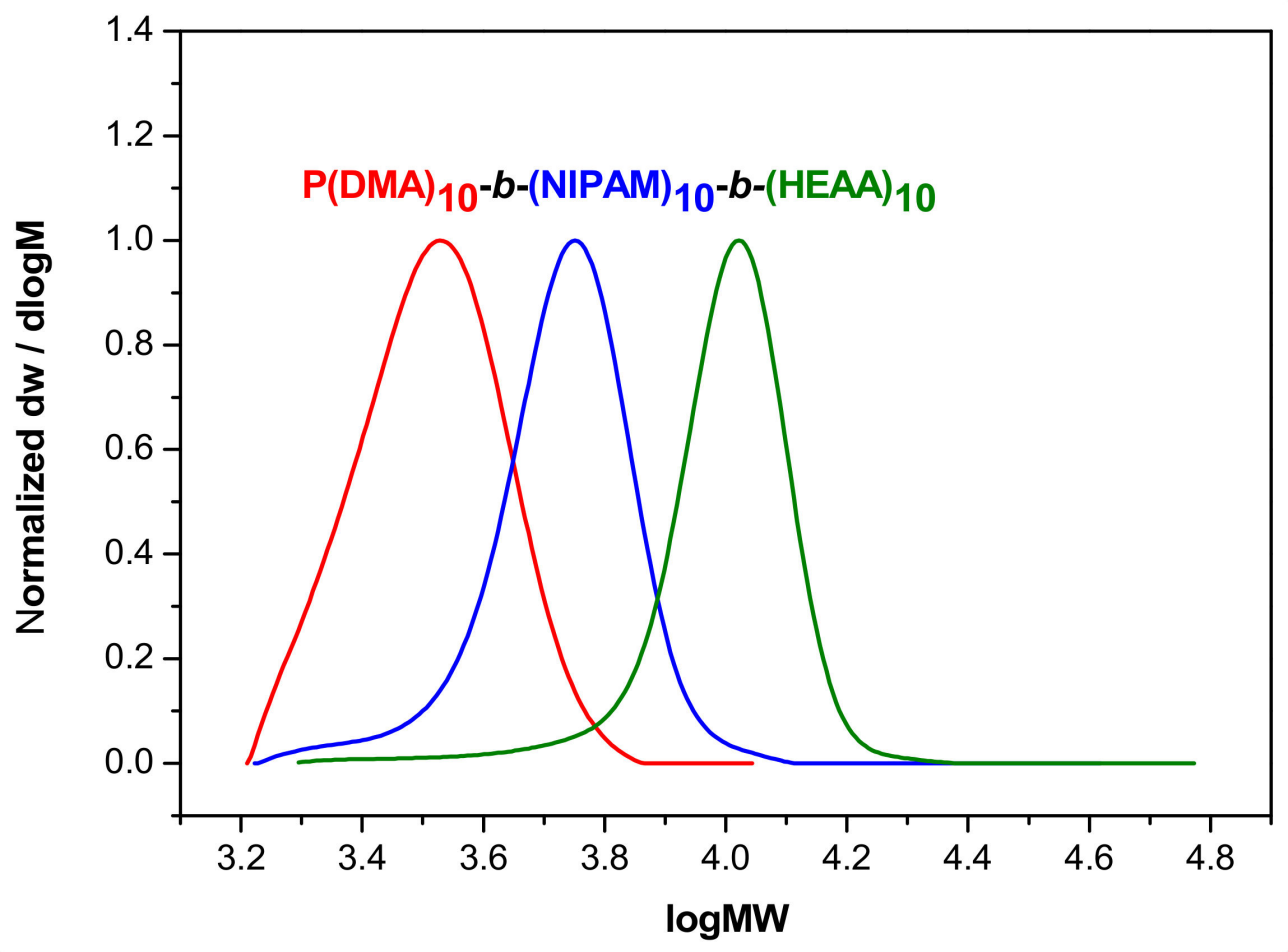
The evolution of molecular weights of successive block extensions of DMA, NIPAM and HEAA by iterative Cu(0)-mediated polymerization.

**Figure 4:**
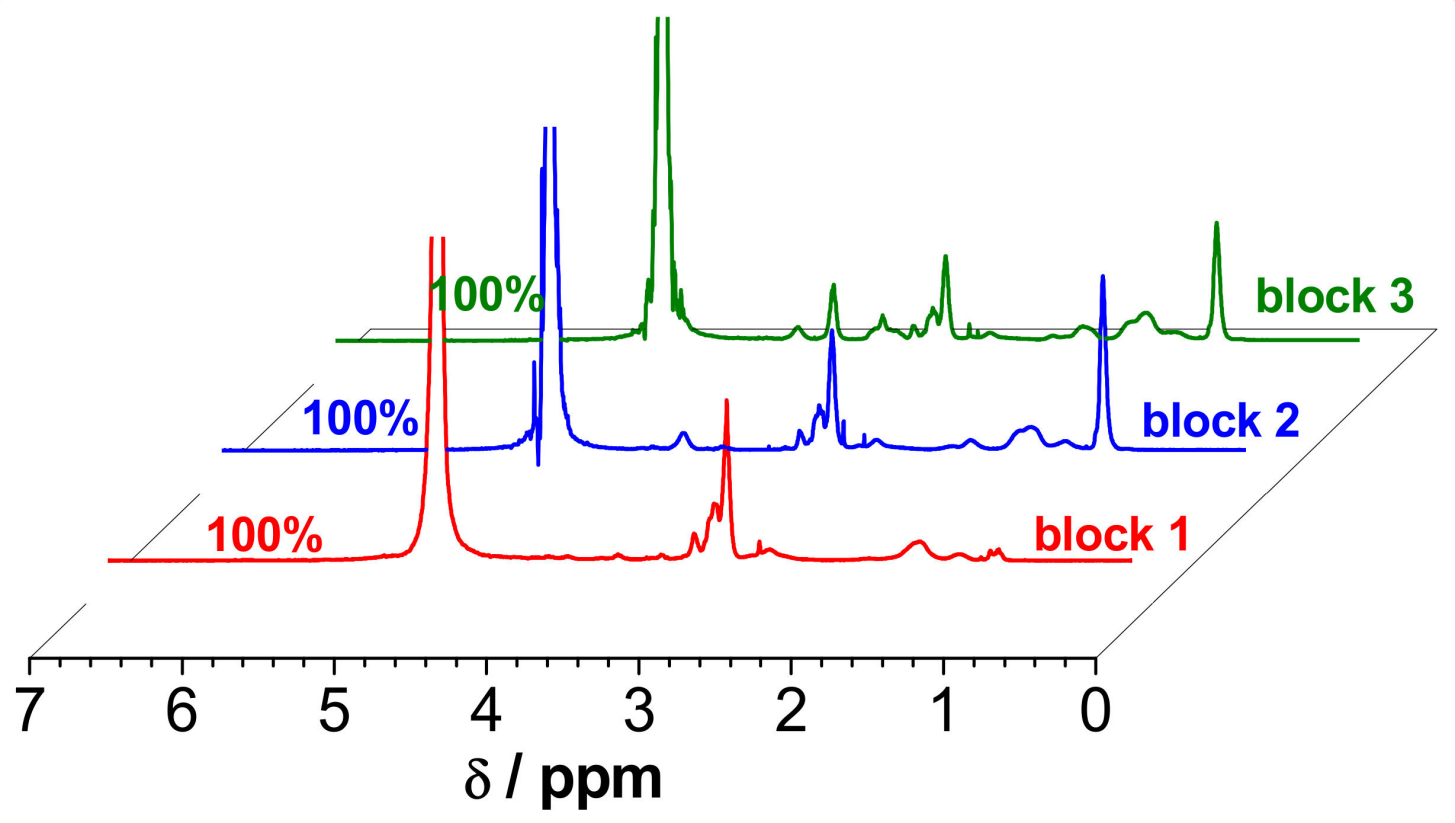
^1^H nuclear magnetic resonance (NMR) spectra (D_2_O) for triblock copolymers of DMA, NIPAM and HEAA and by iterative Cu(0)-mediated polymerization.

In line with the PHEAA macroinitiator investigation, DMA was also chosen to initiate the triblock copolymer (Scheme 2). Similarly, fast, controlled polymerization was achieved (less than 1 h) (Table 2), while the molecular weight distributions were narrow over block copolymerization. Moreover, ^1^H NMR and SEC confirmed the successive chain extensions, and narrow dispersities were retained throughout (final Ð = 1.08) (Figure 3 and Figure 4), implying the potential for precise control over discrete monomer sequences within the final polymer composition. In terms of the scale-up of this reaction, it is worth conducting more studies with the consideration of optimizing the reaction conditions in future research projects.

### 3.2. Investigating the Potential for Utilizing Halogen Exchange to Improve Resistance against Hydrolysis 

**Scheme 3:**
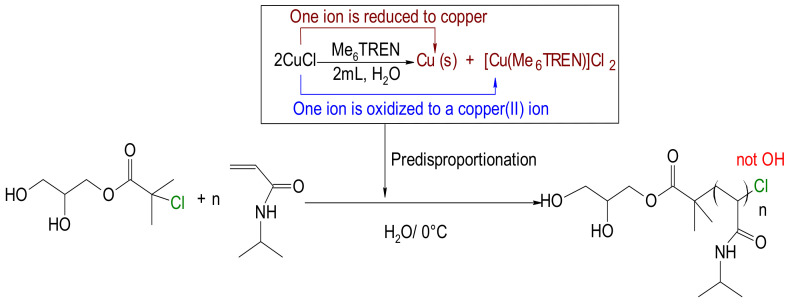
Synthesis of PNIPAM by Cu(0)-mediated polymerization in H_2_O at 0.0 °C via prior disproportionation of CuCl/ Me_6_TREN. Conditions: [chloro-initiator]:[CuCl]:[Me_6_TREN] = 1:0.4:0.4.

In a previous work [36], hydrolysis was found in a polyacrylamide polymerized at 0.0 °C using 600 MHz NMR, and it was concluded that the hydrolysis of bromine is the predominant termination reaction. In addition, the hydrolysis reaction was found to be more obvious subsequent to full conversion, but lower reaction temperatures suppress the rate. It is well known that the alkyl halides (R-X) reactivities follow the order I > Br > Cl. Knowledge of the R-X reactivity can be exploited for the selection of appropriate initiators, particularly for the efficient synthesis of block copolymers [44]. To stabilize the carbon–halogen bond against facial hydrolysis, the bromo-based initiator/CuBr system was replaced with a chloro-based initiator/CuCl (Scheme 3). The method’s description is similar to that in Section 2.3.

**Scheme 4:**
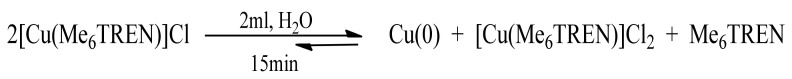
Disproportionation of CuCl in the presence of Me_6_TREN. Conditions: [CuCl]:[Me_6_TREN] = 1:1 in 2 mL H_2_O.

**Figure 5:**
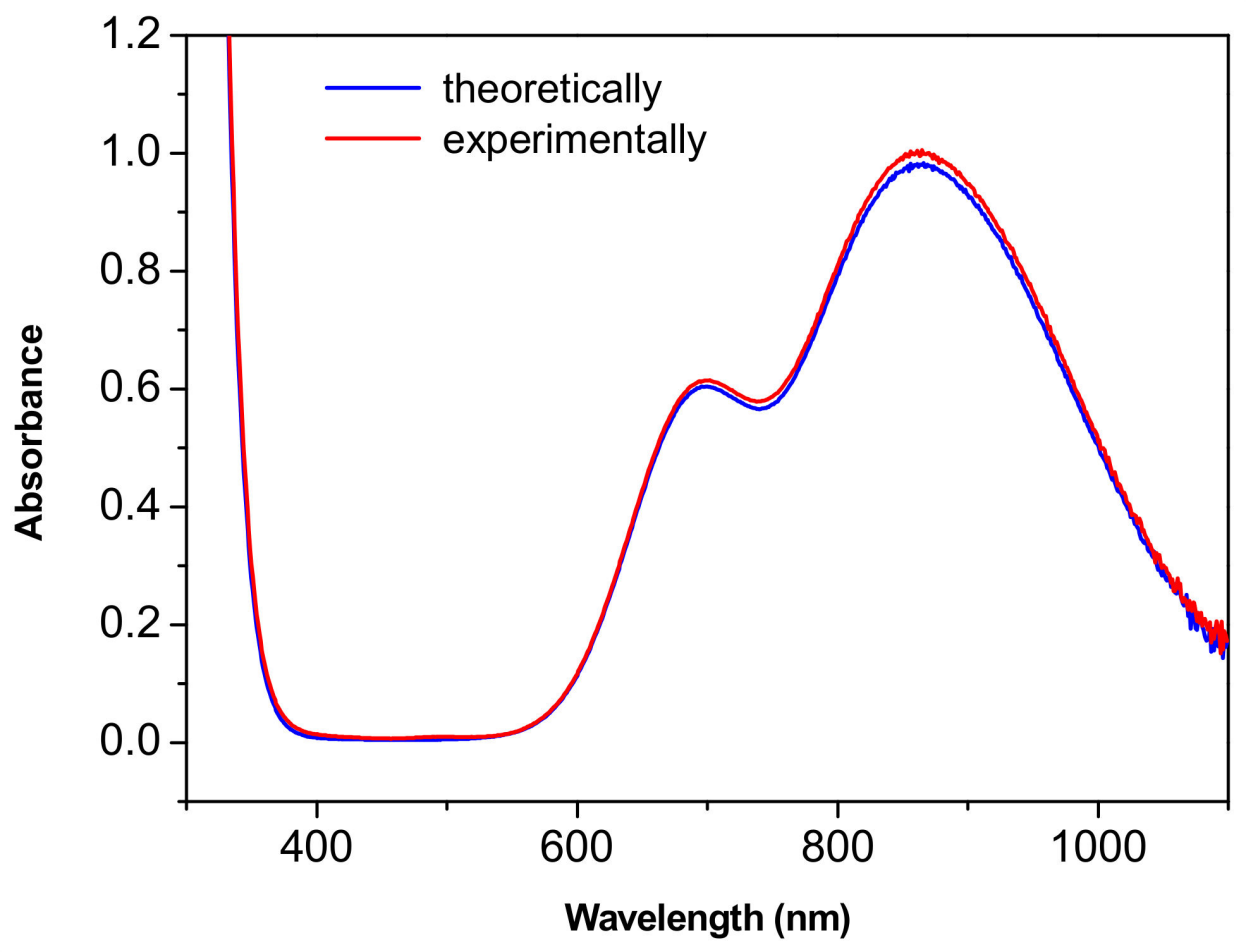
UV–vis spectra of CuCl_2_ solution in the presence of Me_6_TREN in 2 mL of H_2_O. The red line represents the UV–vis spectrum of the disproportionation of CuCl (conditions: [CuCl]:[Me_6_TREN] = 1:1 in 2 mL H_2_O). The sample was diluted before analysis into degassed H_2_O.

The disproportionation of CuCl into Cu(0) and [Cu(Me_6_TREN)]Cl_2_ needed to be examined prior to using CuCl as a catalyst (Scheme 4). Disproportionation was monitored by UV–vis absorbance (λmax ~900 nm) corresponding to the in situ-generated [Cu(Me_6_TREN)]Cl_2_ complex and was recorded to be ~100% (Figure 5).

**Figure 6:**
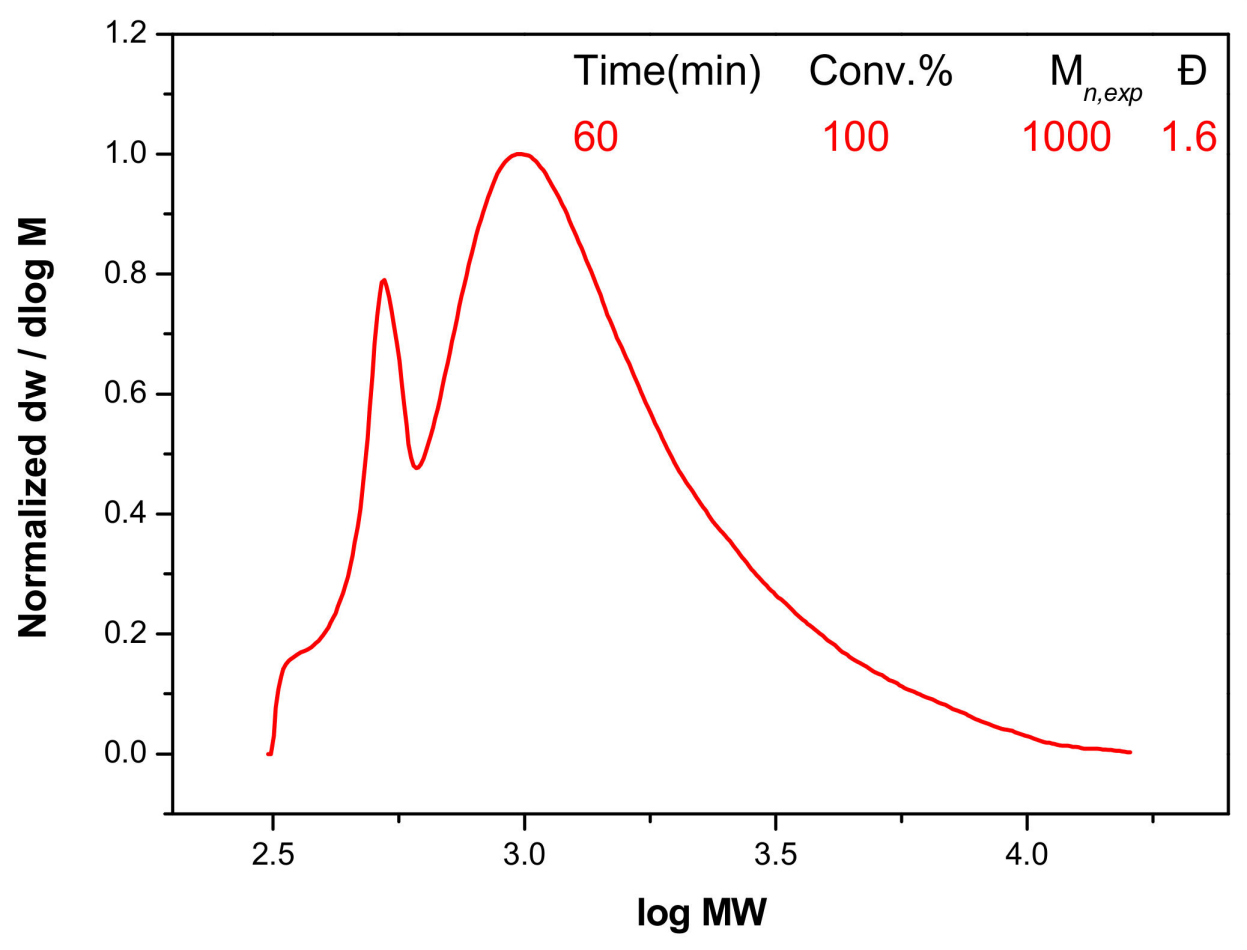
DMF SEC molecular weight distributions for PNIPAM via Cu(0)-mediated polymerization in H_2_O at 0.0 °C. [chloro-initiator]:[CuCl]:[Me_6_TREN] = 1:0.4:0.4.

**Figure 7:**
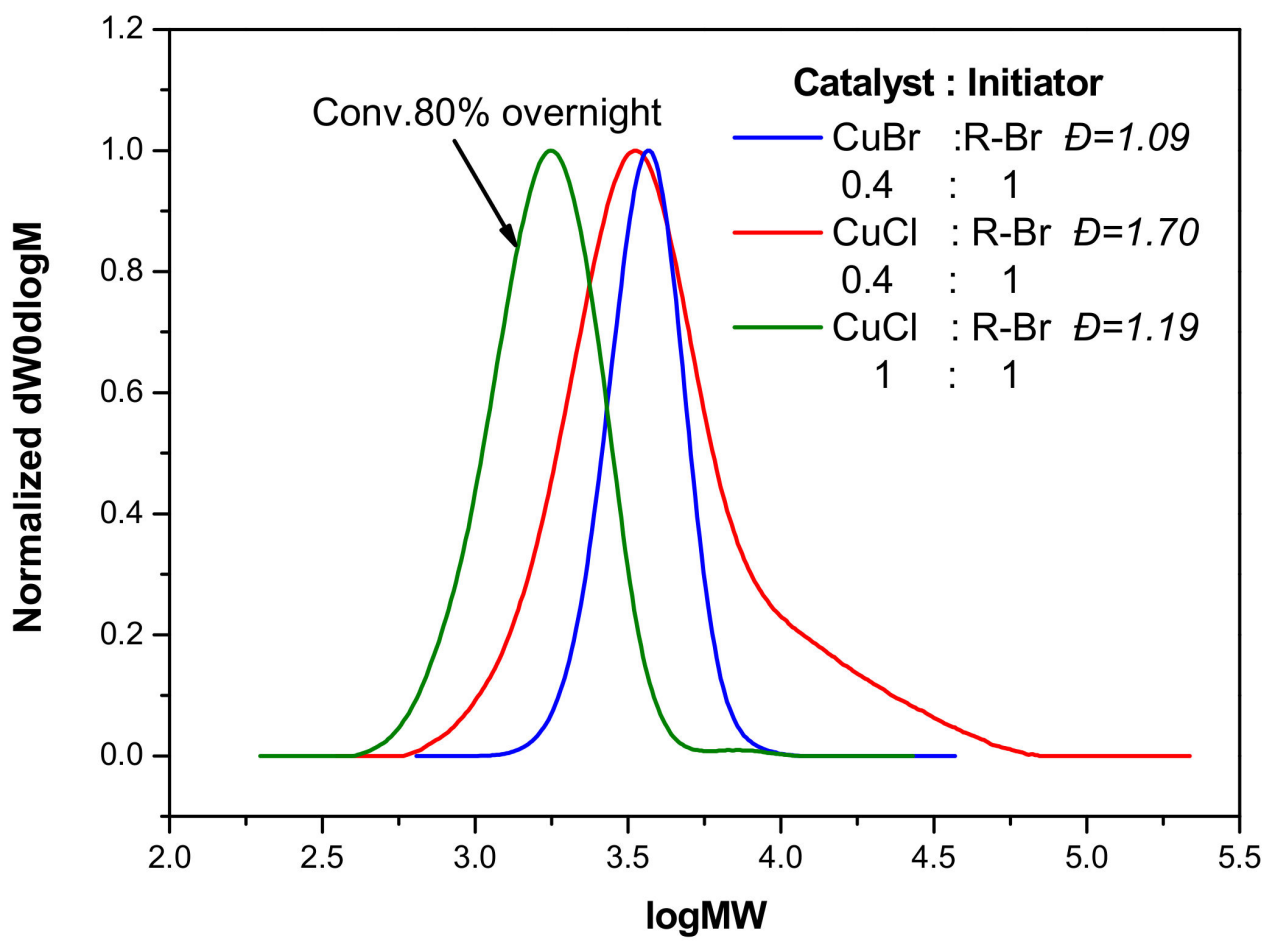
DMF SEC molecular weight distributions for PNIPAM via Cu(0)-mediated polymerization in H_2_O at 0.0 °C (blue trace) [bromo-initiator]:[CuBr]:[Me_6_TREN] = 1:0.4:0.4. and (red trace) [bromo-initiator]:[CuCl]:[Me_6_TREN] = 1:0.4:0.4. (green trace) [bromo-initiator]:[CuCl]:[Me_6_TREN] = 1:1:1.

Utilizing the same ratio [chloro-initiator]:[CuCl]:[Me_6_TREN] = 1:0.4:0.4 and employing NIPAM as a model acrylamide in H_2_O at 0.0 °C resulted in a slower polymerization rate (100% conversion, within 60 min not 11 min) [38] in comparison with the bromo-initiator/CuBr and broad MWDs (Ð = 1.60) (Figure 6 and Figure 7, blue trace). The slow polymerization rate was expected, as the R-Cl was less active than R-Br, but the broad dispersity could be attributed to either an inefficient initiation step or/and the stronger Cu(II)–Cl_2_ bond. 

**Scheme 5:**
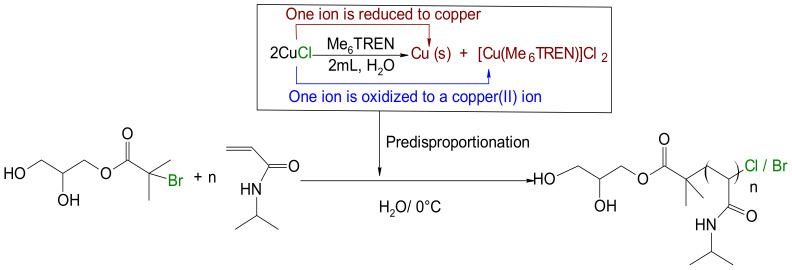
Synthesis of PNIPAM by Cu(0)-mediated polymerization in H_2_O at 0.0 °C via prior disproportionation of [Cu(Me_6_TREN)Cl]. Conditions: [bromo-initiator]:[CuCl]:[Me_6_TREN] = 1:0.4:0.4.

To circumvent this, the halide exchange process was investigated. Halogen exchange during ATRP using mixed halide initiation systems, (R-X/Cu-Y (X, Y = Br, Cl), has been proven to improve the control of ATRP [45,46,47,48,49]. Therefore, the bromo-initiator/CuCl system was employed under the same conditions and procedure that were previously used (Scheme 5). A fast polymerization rate was observed (100% conversion within 22 min) due to the efficient initiation (R-Br); however, the dispersity was still uncontrolled (Figure 7, red trace). Interestingly, when the catalytic ratio was increased to [CuCl]:[Me_6_TREN] = [1]:[1], the polymerization proceeded in a controlled manner (Ð = 1.15) (Figure 7, green trace). However, the monomer conversion was 80%, reflecting the slow polymerization rate. There is a general need to optimize the conditions in the future; nonetheless, these investigations were intended as a feasibility study, and, thus, it is satisfactory that we were able to utilize a mixed halide initiation system of aqueous Cu(0)-mediated polymerization.

## 4. Conclusions

The synthesis of thermosensitive homo-polymers and multiblock copolymers in aqueous media at or below ambient temperature has always been a challenge with respect to the control of the polymerization. This work proposed a simple and highly efficient route to synthesize a well-defined triblock copolymer, model ABC, initiated from PHEAA or utilizing Cu(0)-mediated RDRP in pure water at or below room temperature. A PDMA-macroinitiator was employed to synthesize another triblock copolymer in a similar manner but with a different sequence of monomer addition. The ultrafast one-pot synthesis of sequence-controlled triblock copolymers via iterative sequential monomer addition after full conversion, without any purification steps throughout the monomer additions, was exploited. The successive chain extensions of the block copolymers were demonstrated by SEC analysis, and the compositions of triblock copolymers were confirmed via ^1^H NMR. The robust process showed its suitability for the generation of ABC-type block copolymers with different block sequences from different acrylamides. Moreover, the potential for monomer–copper interactions, which could influence the living nature of the system, was investigated. The bromo-based initiator/CuBr system was switched with a chloro-based initiator/CuCl in order to stabilize the carbon–halogen bond. Finally, we may propose this approach as a potential method for industrial applications that specifically require pure water as the solvent at or below ambient temperature.

## Figures and Tables

**Table 1:** Summary of characteristics of triblock copolymers of HEAA, NIPAM and DMA by iterative Cu(0)-mediated polymerization.

Sequence Distribution	Conv. (%)	Time Per Block (Min)	M_n,th_	M_n,SEC_	Ð
Block 1	100	20	1400	4600	1.09
Block 2	100	40	2500	6500	1.08
Block 3	98	40	3500	9700	1.09

**Table 2:** Summary of characteristics of triblock copolymers of DMA, NIPAM and HEAA by iterative Cu(0)-mediated polymerization.

Sequence Distribution	Conv. (%)	Time Per Block (Min)	M_n,th_	M_n,SEC_	Ð
Block 1	100	2	1200	3100	1.08
Block 2	100	25	2400	5100	1.08
Block 3	100	25	3500	9500	1.08

## Data Availability

The data presented in this study are available on request from the corresponding author. The data are not publicly available due to privacy.
